# 3-Fluoro-*N*-(*p*-tol­yl)benzamide

**DOI:** 10.1107/S160053680803225X

**Published:** 2008-10-11

**Authors:** Aamer Saeed, Rasheed Ahmad Khera, Kazuma Gotoh, Hiroyuki Ishida

**Affiliations:** a Department of Chemistry, Quaid-i-Azam University, Islamabad 45320, Pakistan; b Department of Chemistry, Faculty of Science, Okayama University, Okayama 700-8530, Japan

## Abstract

In the crystal structure of the title compound, C_14_H_12_FNO, the amide –NHCO– mean plane makes dihedral angles of 28.6 (2) and 37.5 (2)° with the mean planes through the fluoro­benzene and methyl­benzene units, respectively. The dihedral angle between the two benzene ring mean planes is 65.69 (10)°. In the crystal structure, mol­ecules are linked through N—H⋯O hydrogen bonds and stack along the *b* axis.

## Related literature

For related structures, see: Chopra & Row (2005 ▶); Saeed *et al.* (2008 ▶).
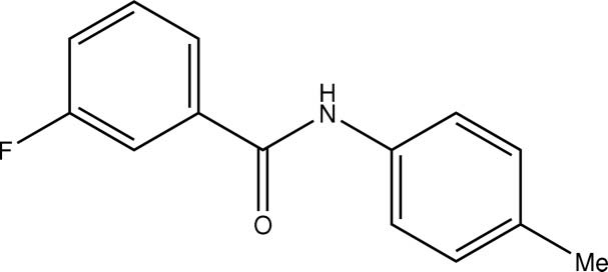


## Experimental

### 

#### Crystal data

C_14_H_12_FNO
*M* *_r_* = 229.25
Monoclinic, 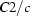

*a* = 27.645 (3) Å
*b* = 5.2618 (6) Å
*c* = 15.892 (2) Å
β = 93.519 (3)°
*V* = 2307.3 (5) Å^3^
*Z* = 8
Mo *K*α radiation
μ = 0.09 mm^−1^
*T* = 223 (1) K
0.40 × 0.35 × 0.18 mm

#### Data collection

Rigaku R-AXIS RAPIDII diffractometer
Absorption correction: numerical (**ABSCOR**; Higashi, 1999 ▶) *T*_min_ = 0.968, *T*_max_ = 0.983
13860 measured reflections
3357 independent reflections
1779 reflections with *I* > 2σ(*I*)
*R*_int_ = 0.055

#### Refinement

*R*[*F* ^2^ > 2σ(*F* ^2^)] = 0.073
*wR*(*F* ^2^) = 0.240
*S* = 1.01
3357 reflections
158 parameters
H atoms treated by a mixture of independent and constrained refinement
Δρ_max_ = 0.32 e Å^−3^
Δρ_min_ = −0.21 e Å^−3^

### 

Data collection: *PROCESS-AUTO* (Rigaku/MSC, 2004 ▶); cell refinement: *PROCESS-AUTO*; data reduction: *CrystalStructure* (Rigaku/MSC, 2004 ▶); program(s) used to solve structure: *SHELXS97* (Sheldrick, 2008 ▶); program(s) used to refine structure: *SHELXL97* (Sheldrick, 2008 ▶); molecular graphics: *ORTEP-3* (Farrugia, 1997 ▶); software used to prepare material for publication: *CrystalStructure* and *PLATON* (Spek, 2003 ▶).

## Supplementary Material

Crystal structure: contains datablocks global, I. DOI: 10.1107/S160053680803225X/su2064sup1.cif

Structure factors: contains datablocks I. DOI: 10.1107/S160053680803225X/su2064Isup2.hkl

Additional supplementary materials:  crystallographic information; 3D view; checkCIF report

## Figures and Tables

**Table 1:** Hydrogen-bond geometry (Å, °)

*D*—H⋯*A*	*D*—H	H⋯*A*	*D*⋯*A*	*D*—H⋯*A*
N1—H1⋯O1^i^	0.77 (2)	2.35 (2)	3.087 (3)	161 (2)

Symmetry code: (i) 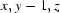.

**Fig. 1.**
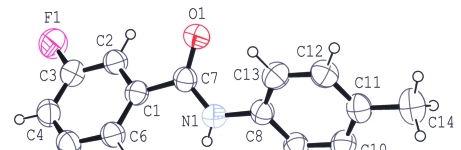
A view of the molecular structure of the title compound. The displacement ellipsoids are drawn at the 40% probability level.

**Fig. 2.**
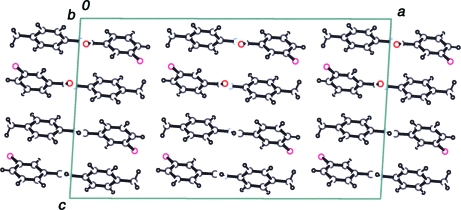
A view along the b axis of the crystal packing of the title compound.

## supplementary crystallographic information

### Comment

The background to this study has been described in our earlier paper on 4-chloro-*N*-(2-chlorophenyl)-benzamide (Saeed *et al.*, 2008).

In the crystal structure of the title compound the two benzene rings are considerably twisted with respect to one another, with a dihedral angle of 65.69 (10)°. The amide –NHCO– mean plane makes dihedral angles of 28.6 (2) and 37.5 (2)° with the best mean planes through the fluorobenzene and methylbenzene units, respectively. In the crystal the molecules are linked through N—H···O hydrogen bonds and stack up the *b* axis.

No C—H···F hydrogen bonds were observed here, in contrast to the situation in 4-fluoro-*N*-(2-fluorophenyl)-benzamide (Chopra & Row, 2005).

### Experimental

4-Fluorobenzoyl chloride (5.4 mmol) in CHCl_3_ was treated with 4-methylaniline (21.6 mmol) under a nitrogen atmosphere at reflux for 4 h. Upon cooling the reaction mixture was diluted with CHCl_3_ and washed consecutively with aq 1 *M* HCl and saturated aq NaHCO_3_. The organic layer was dried over anhydrous sodium sulfate and concentrated under reduced pressure. Crystallization of the residue in CHCl_3_ afforded the title compound (84%) as white needles: Anal. calcd. for C_14_H_12_FNO: C 73.35, H 5.28, N 6.11%; found: C 73.30, H 5.32, N 6.09%.

### Refinement

The N-bound H atom was located in a difference Fourier map and was freely refined. The other H atoms were positioned geometrically (C—H = 0.94 and 0.97 Å) and treated as riding atoms, with *U*_iso_(H) = 1.2*U*_eq_(C) or 1.5*U*_eq_(methyl C).

### Crystal data

**Table:**

C_14_H_12_FNO	*F*(000) = 960.00
*M**_r_* = 229.25	*D*_x_ = 1.320 Mg m^−^^3^
Monoclinic, *C*2/*c*	Mo *K*α radiation, λ = 0.71075 Å
Hall symbol: -C 2yc	Cell parameters from 7127 reflections
*a* = 27.645 (3) Å	θ = 3.0–30.0°
*b* = 5.2618 (6) Å	µ = 0.09 mm^−^^1^
*c* = 15.892 (2) Å	*T* = 223 K
β = 93.519 (3)°	Block, colorless
*V* = 2307.3 (5) Å^3^	0.40 × 0.35 × 0.18 mm
*Z* = 8	

### Data collection

**Table:**

Rigaku R-AXIS RAPIDII diffractometer	1779 reflections with *I* > 2σ(*I*)
Detector resolution: 10.00 pixels mm^-1^	*R*_int_ = 0.055
ω scans	θ_max_ = 30.0°
Absorption correction: numerical (*ABSCOR*; Higashi, 1999)	*h* = −38→38
*T*_min_ = 0.968, *T*_max_ = 0.983	*k* = −6→7
13860 measured reflections	*l* = −22→22
3357 independent reflections	

### Refinement

**Table:**

Refinement on *F*^2^	Primary atom site location: structure-invariant direct methods
Least-squares matrix: full	Secondary atom site location: difference Fourier map
*R*[*F*^2^ > 2σ(*F*^2^)] = 0.073	Hydrogen site location: inferred from neighbouring sites
*wR*(*F*^2^) = 0.240	H atoms treated by a mixture of independent and constrained refinement
*S* = 1.01	*w* = 1/[σ^2^(*F*_o_^2^) + (0.1335*P*)^2^] where *P* = (*F*_o_^2^ + 2*F*_c_^2^)/3
3357 reflections	(Δ/σ)_max_ = 0.001
158 parameters	Δρ_max_ = 0.32 e Å^−^^3^
0 restraints	Δρ_min_ = −0.20 e Å^−^^3^

### Special details

**Table:**

Geometry. All e.s.d.'s (except the e.s.d. in the dihedral angle between two l.s. planes) are estimated using the full covariance matrix. The cell e.s.d.'s are taken into account individually in the estimation of e.s.d.'s in distances, angles and torsion angles; correlations between e.s.d.'s in cell parameters are only used when they are defined by crystal symmetry. An approximate (isotropic) treatment of cell e.s.d.'s is used for estimating e.s.d.'s involving l.s. planes.
Refinement. Refinement of *F*^2^ against ALL reflections. The weighted *R*-factor *wR* and goodness of fit *S* are based on *F*^2^, conventional *R*-factors *R* are based on *F*, with *F* set to zero for negative *F*^2^. The threshold expression of *F*^2^ > σ(*F*^2^) is used only for calculating *R*-factors(gt) *etc*. and is not relevant to the choice of reflections for refinement. *R*-factors based on *F*^2^ are statistically about twice as large as those based on *F*, and *R*- factors based on ALL data will be even larger.

### Fractional atomic coordinates and isotropic or equivalent isotropic displacement parameters (Å^2^)

**Table:**

	*x*	*y*	*z*	*U*_iso_*/*U*_eq_	
F1	0.69906 (5)	0.5995 (3)	0.23465 (10)	0.0965 (5)	
O1	0.52251 (5)	0.6096 (3)	0.14071 (11)	0.0781 (5)	
N1	0.50521 (6)	0.1882 (4)	0.12722 (11)	0.0639 (5)	
C1	0.58887 (6)	0.3226 (4)	0.14164 (11)	0.0594 (5)	
C2	0.62009 (7)	0.4878 (4)	0.18591 (12)	0.0656 (5)	
H2	0.6081	0.6327	0.2122	0.079*	
C3	0.66868 (8)	0.4361 (4)	0.19066 (14)	0.0703 (5)	
C4	0.68819 (7)	0.2292 (5)	0.15293 (14)	0.0745 (6)	
H4	0.7217	0.1984	0.1576	0.089*	
C5	0.65712 (7)	0.0677 (4)	0.10794 (14)	0.0732 (6)	
H5	0.6697	−0.0741	0.0807	0.088*	
C6	0.60772 (7)	0.1105 (4)	0.10214 (12)	0.0657 (5)	
H6	0.5869	−0.0026	0.0718	0.079*	
C7	0.53603 (7)	0.3860 (4)	0.13667 (11)	0.0608 (5)	
C8	0.45358 (7)	0.2026 (4)	0.11934 (11)	0.0599 (5)	
C9	0.42706 (7)	0.0146 (4)	0.15561 (13)	0.0675 (5)	
H9	0.4431	−0.1164	0.1865	0.081*	
C10	0.37703 (7)	0.0181 (4)	0.14673 (13)	0.0729 (6)	
H10	0.3594	−0.1125	0.1712	0.087*	
C11	0.35222 (7)	0.2093 (4)	0.10264 (11)	0.0675 (5)	
C12	0.37940 (7)	0.3956 (4)	0.06655 (13)	0.0705 (6)	
H12	0.3634	0.5269	0.0359	0.085*	
C13	0.42967 (8)	0.3940 (4)	0.07434 (13)	0.0704 (5)	
H13	0.4474	0.5228	0.0491	0.085*	
C14	0.29774 (8)	0.2111 (6)	0.09397 (16)	0.0916 (8)	
H14A	0.2850	0.2043	0.1495	0.137*	
H14B	0.2864	0.0647	0.0613	0.137*	
H14C	0.2867	0.3655	0.0655	0.137*	
H1	0.5158 (7)	0.055 (4)	0.1353 (12)	0.058 (6)*	

### Atomic displacement parameters (Å^2^)

**Table:**

	*U*^11^	*U*^22^	*U*^33^	*U*^12^	*U*^13^	*U*^23^
F1	0.0752 (8)	0.0995 (10)	0.1131 (11)	−0.0114 (7)	−0.0082 (7)	−0.0173 (8)
O1	0.0711 (9)	0.0616 (9)	0.1023 (12)	0.0027 (7)	0.0107 (7)	−0.0059 (7)
N1	0.0633 (9)	0.0566 (9)	0.0719 (10)	0.0009 (9)	0.0063 (7)	0.0030 (8)
C1	0.0655 (11)	0.0619 (10)	0.0518 (9)	−0.0016 (8)	0.0112 (7)	0.0049 (7)
C2	0.0692 (12)	0.0646 (11)	0.0636 (11)	−0.0002 (10)	0.0087 (8)	−0.0013 (9)
C3	0.0681 (12)	0.0758 (13)	0.0669 (11)	−0.0049 (10)	0.0035 (9)	0.0027 (9)
C4	0.0648 (11)	0.0822 (14)	0.0779 (13)	0.0043 (11)	0.0148 (9)	0.0089 (11)
C5	0.0746 (13)	0.0759 (13)	0.0709 (12)	0.0088 (11)	0.0190 (9)	−0.0006 (10)
C6	0.0705 (11)	0.0665 (11)	0.0610 (10)	0.0004 (9)	0.0127 (8)	−0.0028 (8)
C7	0.0625 (10)	0.0636 (11)	0.0570 (10)	0.0008 (9)	0.0088 (8)	−0.0002 (8)
C8	0.0605 (10)	0.0645 (10)	0.0551 (9)	−0.0013 (8)	0.0076 (7)	−0.0050 (8)
C9	0.0657 (11)	0.0717 (12)	0.0659 (11)	0.0011 (9)	0.0100 (8)	0.0067 (9)
C10	0.0717 (12)	0.0787 (13)	0.0694 (12)	−0.0049 (11)	0.0141 (9)	0.0063 (10)
C11	0.0635 (11)	0.0852 (14)	0.0540 (10)	0.0019 (10)	0.0058 (8)	−0.0085 (9)
C12	0.0712 (12)	0.0729 (13)	0.0663 (12)	0.0063 (10)	−0.0044 (9)	0.0028 (9)
C13	0.0763 (12)	0.0725 (12)	0.0626 (11)	−0.0045 (10)	0.0041 (9)	0.0088 (9)
C14	0.0683 (13)	0.126 (2)	0.0807 (15)	0.0047 (14)	0.0050 (11)	−0.0041 (14)

### Geometric parameters (Å, °)

**Table:**

F1—C3	1.364 (2)	C6—H6	0.9400
O1—C7	1.238 (2)	C8—C9	1.378 (3)
N1—C7	1.347 (3)	C8—C13	1.380 (3)
N1—C8	1.427 (2)	C9—C10	1.382 (3)
N1—H1	0.77 (2)	C9—H9	0.9400
C1—C2	1.386 (3)	C10—C11	1.384 (3)
C1—C6	1.397 (3)	C10—H10	0.9400
C1—C7	1.496 (3)	C11—C12	1.381 (3)
C2—C3	1.368 (3)	C11—C14	1.504 (3)
C2—H2	0.9400	C12—C13	1.388 (3)
C3—C4	1.370 (3)	C12—H12	0.9400
C4—C5	1.377 (3)	C13—H13	0.9400
C4—H4	0.9400	C14—H14A	0.9700
C5—C6	1.382 (3)	C14—H14B	0.9700
C5—H5	0.9400	C14—H14C	0.9700
			
C7—N1—C8	126.25 (17)	C9—C8—C13	119.37 (18)
C7—N1—H1	117.0 (15)	C9—C8—N1	118.68 (17)
C8—N1—H1	115.6 (15)	C13—C8—N1	121.91 (17)
C2—C1—C6	119.43 (17)	C8—C9—C10	120.14 (19)
C2—C1—C7	117.56 (17)	C8—C9—H9	119.9
C6—C1—C7	122.99 (17)	C10—C9—H9	119.9
C3—C2—C1	118.83 (19)	C9—C10—C11	121.58 (19)
C3—C2—H2	120.6	C9—C10—H10	119.2
C1—C2—H2	120.6	C11—C10—H10	119.2
F1—C3—C2	118.34 (19)	C12—C11—C10	117.44 (17)
F1—C3—C4	118.61 (19)	C12—C11—C14	121.6 (2)
C2—C3—C4	123.05 (19)	C10—C11—C14	120.9 (2)
C3—C4—C5	117.92 (19)	C11—C12—C13	121.75 (18)
C3—C4—H4	121.0	C11—C12—H12	119.1
C5—C4—H4	121.0	C13—C12—H12	119.1
C4—C5—C6	121.1 (2)	C8—C13—C12	119.71 (18)
C4—C5—H5	119.4	C8—C13—H13	120.1
C6—C5—H5	119.4	C12—C13—H13	120.1
C5—C6—C1	119.65 (19)	C11—C14—H14A	109.5
C5—C6—H6	120.2	C11—C14—H14B	109.5
C1—C6—H6	120.2	H14A—C14—H14B	109.5
O1—C7—N1	123.30 (18)	C11—C14—H14C	109.5
O1—C7—C1	120.43 (17)	H14A—C14—H14C	109.5
N1—C7—C1	116.27 (17)	H14B—C14—H14C	109.5
			
C6—C1—C2—C3	−0.9 (3)	C2—C1—C7—N1	−152.62 (17)
C7—C1—C2—C3	−179.24 (17)	C6—C1—C7—N1	29.1 (3)
C1—C2—C3—F1	−179.77 (18)	C7—N1—C8—C9	−144.0 (2)
C1—C2—C3—C4	0.6 (3)	C7—N1—C8—C13	38.1 (3)
F1—C3—C4—C5	−179.27 (19)	C13—C8—C9—C10	0.1 (3)
C2—C3—C4—C5	0.3 (3)	N1—C8—C9—C10	−177.82 (17)
C3—C4—C5—C6	−1.0 (3)	C8—C9—C10—C11	−0.8 (3)
C4—C5—C6—C1	0.8 (3)	C9—C10—C11—C12	0.9 (3)
C2—C1—C6—C5	0.2 (3)	C9—C10—C11—C14	−179.65 (19)
C7—C1—C6—C5	178.47 (18)	C10—C11—C12—C13	−0.5 (3)
C8—N1—C7—O1	0.9 (3)	C14—C11—C12—C13	−179.9 (2)
C8—N1—C7—C1	−178.66 (15)	C9—C8—C13—C12	0.3 (3)
C2—C1—C7—O1	27.8 (3)	N1—C8—C13—C12	178.19 (17)
C6—C1—C7—O1	−150.5 (2)	C11—C12—C13—C8	−0.1 (3)

### Hydrogen-bond geometry (Å, °)

**Table:**

*D*—H···*A*	*D*—H	H···*A*	*D*···*A*	*D*—H···*A*
N1—H1···O1^i^	0.77 (2)	2.35 (2)	3.087 (3)	161 (2)

Symmetry codes: (i) *x*, *y*−1, *z*.
